# Comparison of Dysautonomia Across Species: Current Knowledge and Future Research Opportunities

**DOI:** 10.1111/jvim.70140

**Published:** 2025-06-17

**Authors:** Callum N. Atkins, Caroline N. Hahn, Bruce C. McGorum

**Affiliations:** ^1^ Royal (Dick) School of Veterinary Studies The University of Edinburgh Roslin UK

**Keywords:** autonomic nervous system, chromatolysis, dysautonomia, grass sickness, Key‐Gaskell syndrome

## Abstract

Primary dysautonomia is a complex and often fatal autonomic nervous system disease. This literature review consolidates information on dysautonomia across species. Electronic databases (PubMed, Google Scholar and the Equine Grass Sickness Fund website) were systematically searched for veterinary and human medical literature on the topic. Nine hundred and fifty‐six articles were identified, of which 158 were included in this review. The review covers the clinical presentation, etiology, diagnostic approaches, treatment strategies, and prognosis across different species. By integrating findings from multiple species, we aim to enhance understanding and inform future research and medical strategies regarding dysautonomia.

**Abbreviations**
AED: abomasal emptying defect
CD: canine dysautonomia
CGS: chronic grass sickness
CN: cranial nerve
ED: equine dysautonomia
EGS: equine grass sickness
FD: feline dysautonomia
LMN: lower motor neuron
PLR: pupillary light reflex

## 1 Introduction

Primary dysautonomia is a rare neurodegenerative disorder of unknown etiology. Similar clinicopathological features have been reported in various unrelated animal species including dogs, cats, horses, zebras, llamas, alpacas, rabbits, and hares [1, 2, 3, 4, 5, 6, 7, 8, 9]. The predominant clinical findings are attributable to paralysis of the entire gastrointestinal tract caused by severe enteric neuropathy. Definitive diagnosis requires histological demonstration of the pathognomonic chromatolytic degeneration of the neurons of autonomic ganglia, ventral horns of the spinal cord, and brainstem nuclei, with minimal inflammation [10, 11, 12, 13]. Despite substantial advancements in understanding the condition, it remains a challenging and poorly understood veterinary neurological disorder. No effective treatment is available and the prognosis is poor. A common etiological agent is suspected. Ingestion and intestinal absorption of a putative neurotoxin is considered most likely. Collaborative research efforts and continued case reporting are crucial to identify common features and further our understanding of this enigmatic condition. This literature review aims to summarize existing knowledge, compare the condition across species, and promote future research.

## 2 Methods

For the veterinary literature, we systematically searched two electronic databases (PubMed and Google Scholar) between the years 1920 and 2025. Search terms included “equine dysautonomia”, “equine grass sickness”, “feline dysautonomia”, “Key‐Gaskell syndrome”, “canine dysautonomia”, “leporine dysautonomia”, “camelid dysautonomia”, “alpaca dysautonomia”, “llama dysautonomia”, “ovine dysautonomia”, “abomasal emptying defect”, “ovine abomasal impaction”, “bovine dysautonomia”, “calves dysautonomia” and “bovine chromatolysis”. PubMed filters included: publication date “from 1920 to 2025”, text availability “abstract”, article language “English”, and species “other animals”. Google Scholar filters included: custom range “1920–2025” and the “allintitle:” function. Additionally, we reviewed the literature available in the research section of the Equine Grass Sickness Fund website. For the human medical literature, we systematically searched the PubMed electronic database between the years 2020 and 2025 for the term “pure autonomic failure” using the following filters: publication date “five years”, text availability “abstract”, article language “English”, article type “review” and species “human”. The reference lists of the eligible articles included after the electronic searches were also manually searched. The last date these databases were searched was 31st January 2025.

Titles and abstracts were independently reviewed by one of the authors (CA) and the full text of potentially relevant studies was independently reviewed for final inclusion (CA). We eliminated studies with no full text available unless print copies were available for review in the veterinary library of the University of Edinburgh. The final manuscript and list of referenced studies were reviewed independently by all authors.

## 3 Results

**FIGURE 1:**
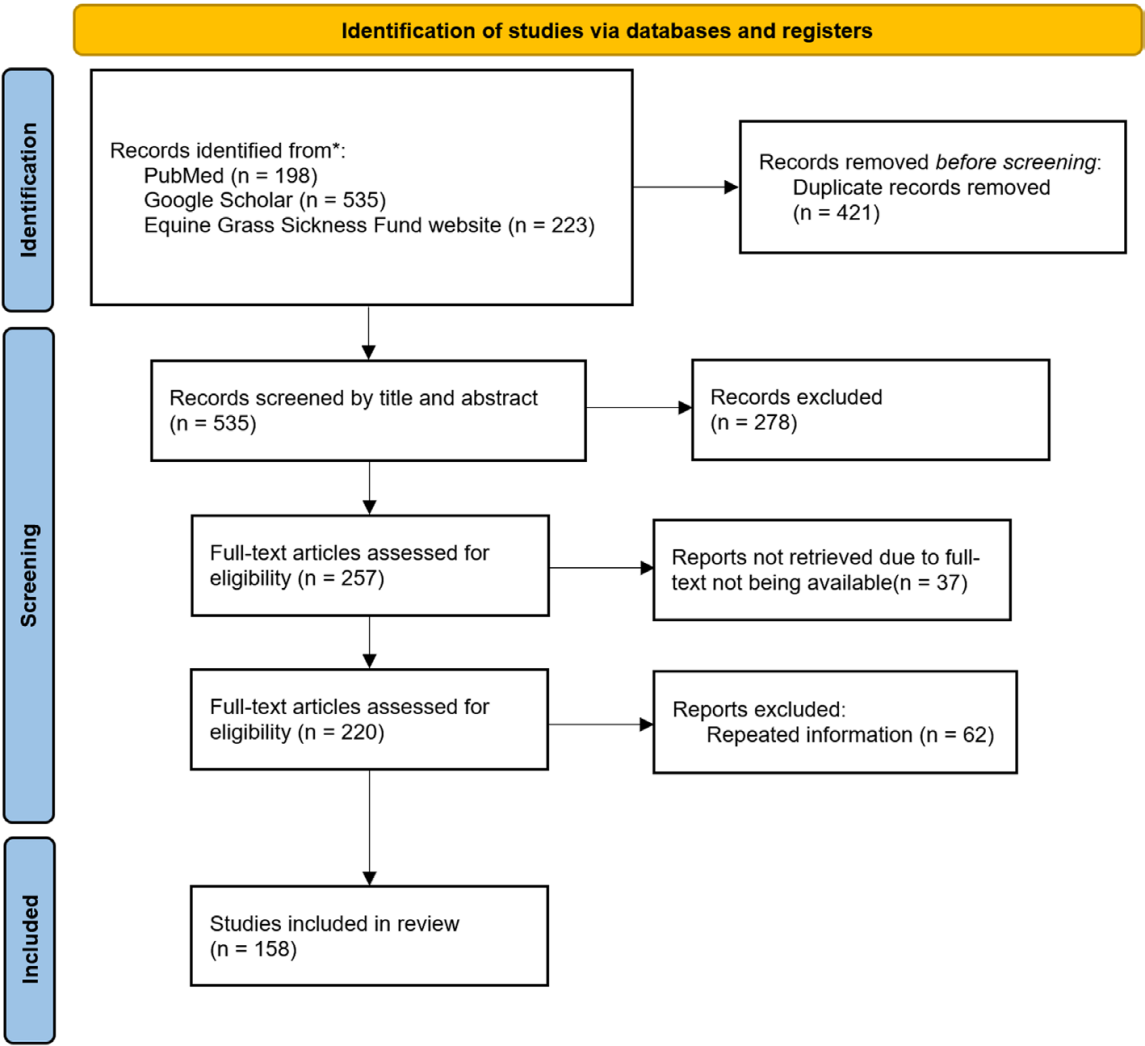
Identification of studies. Reporting items for the systematic literature review (adapted from the Preferred Reporting Items for Systematic Reviews (PRISMA) statement).

The search identified 956 articles. After the application of exclusion criteria, we narrowed this number to 158 articles relevant to the present literature review (Figure 1).

## 4 Equine Dysautonomia

Equine dysautonomia (ED), also known as equine grass sickness (EGS) [1], is a rare and severe neurological disorder that affects the autonomic nervous system and enteric nervous system in grazing horses [14]. It was first identified in Scotland in 1907 [1] and has since been confirmed in many other northern European countries [15, 16, 17, 18]. Suspected cases have been reported in horses in the Falkland Islands [19], Australia [20] and the Patagonia region of South America [21, 22, 23, 24] as well as one mule in North America [25]. The disease does not appear to be limited to domestic equids and has been reported in Prezewalski's horses [26] and zebra [4].

Equine dysautonomia is primarily a disease of young, mature horses kept at grass. It is strongly associated with particular premises, and outbreaks are common [27] with certain pastures having a disproportionately high association with case occurrences [28]. Transmission with the serum of affected acute cases inducing asymptomatic classical disease‐associated pathology in recipient ponies has been demonstrated [29]. A seasonal pattern of ED has been reported, with affected horses identified most commonly in late spring and early summer [30]. The geographic and temporal clustering of cases [27] may reflect climatic influences on etiologic agent exposure [28, 31].

**FIGURE 2:**
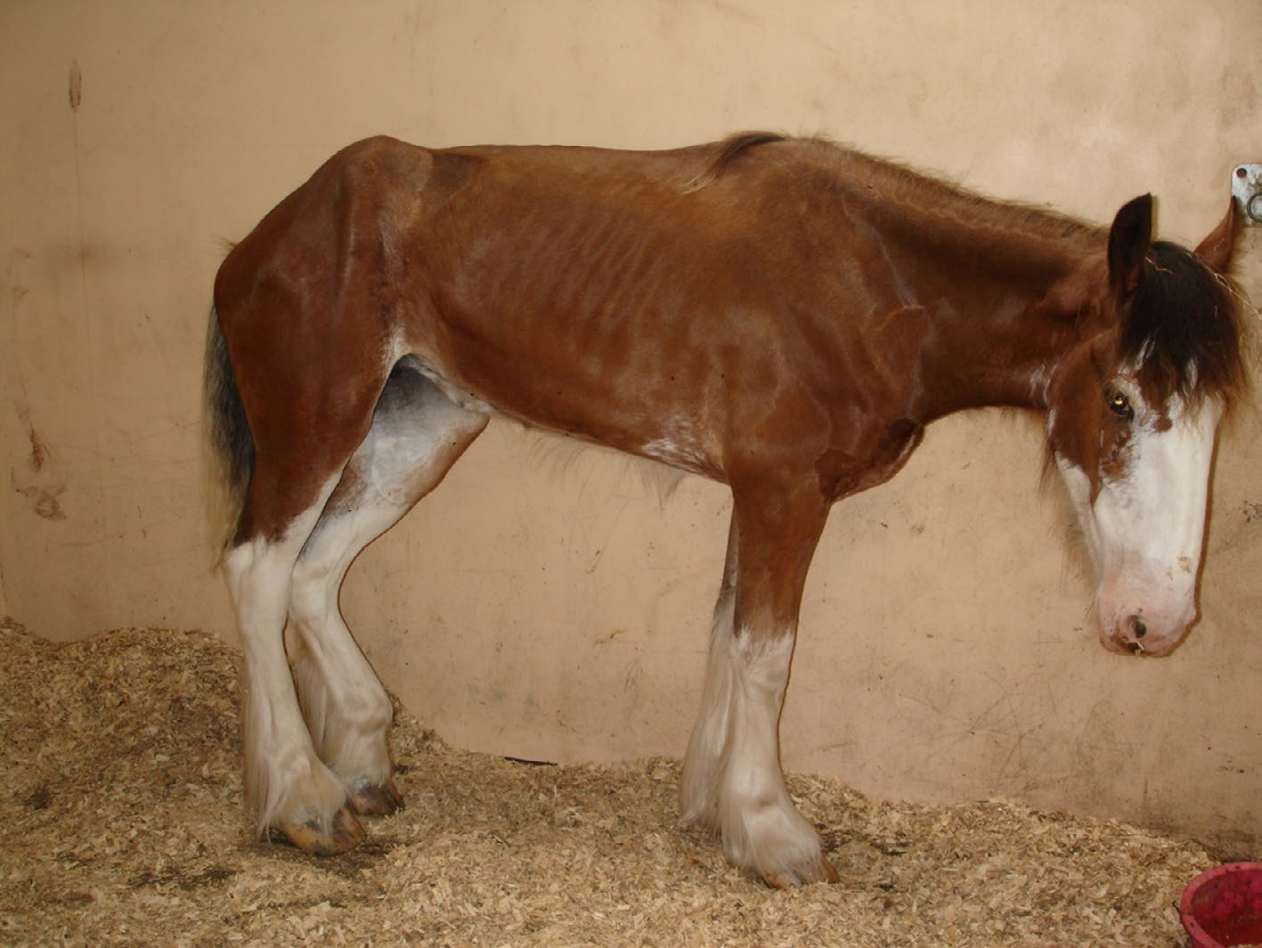
Equine dysautonomia. Horse affected with chronic grass sickness showing the characteristic ‘tucked up’ abdominal silhouette and a base‐narrow stance.

Classical signs include dysphagia and intestinal stasis accompanied by any of many other concurrent abnormalities including tachycardia, ptosis, patchy sweating, muscle fasciculations, abdominal pain, abdominal distension, hypersalivation, pyrexia, narrow‐base stance (Figure 2), paraphimosis, aspiration pneumonia, distended small intestinal loops, megaesophagus, cecal and colonic impaction, and rhinitis sicca [17]. Clinical presentation reflects the severity of neuronal loss with three broadly overlapping phenotypes recognized: acute grass sickness (AGS; severe), subacute grass sickness (SGS; moderate) and chronic grass sickness (CGS; mild) [32]. Acute and subacute cases invariably result in euthanasia [32] whereas up to 50% of chronic cases may survive [33, 34]. Rapid and extensive body weight loss is predictive of outcome for CGS, with non‐survivors reported to lose body weight more rapidly [34]. Treatment of CGS is predominantly nursing care and should be reserved for cases retaining an ability to swallow, a continued interest in food, and absent continuous abdominal pain [17]. After clinical recovery, long‐term complications such as persistent low‐grade dysphagia, recurrent esophageal obstruction, gastric ulceration, episodic colic, and coat changes can be expected [33, 35, 36, 37].

**FIGURE 3:**
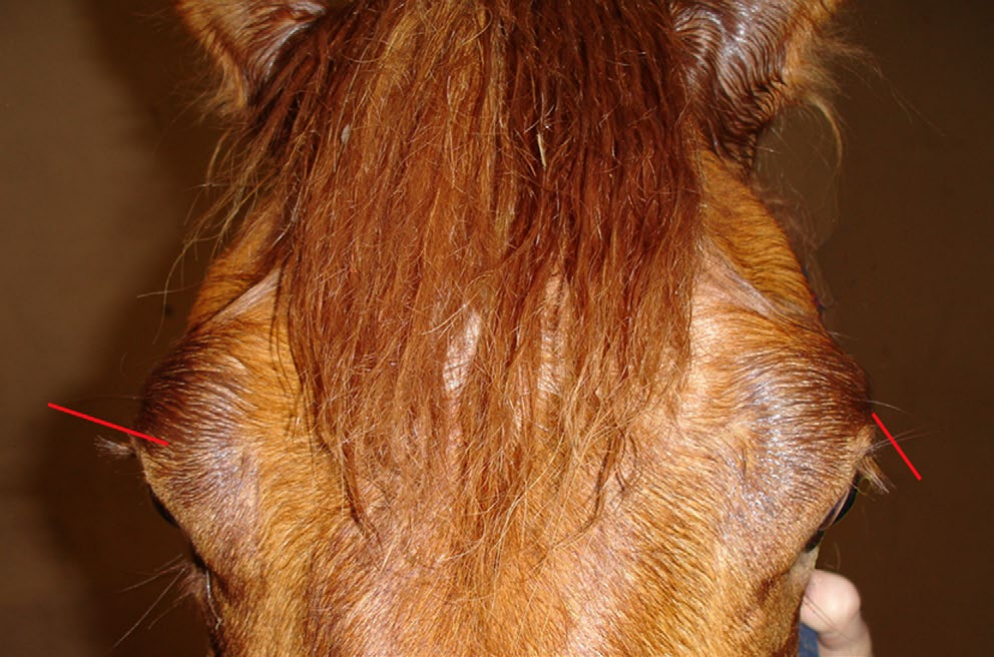
Equine dysautonomia. Equine grass sickness showing the characteristic decrease in angle of the eyelashes (right side of image) and after diagnostic application of 0.5 mL of 0.5% phenylephrine (left side of image).

Diagnostic tests that can be used in suspected cases of ED include the identification of non‐specific ED‐associated hematologic, biochemical, urinary, physiological, and bacteriologic abnormalities; identification of autonomic nervous system or enteric nervous system dysfunction or both; identification of other nervous system dysfunction; and histopathologic evidence of neuropathology in ante‐mortem ileal, rectal, or gustatory papillae biopsy samples [38, 39, 40, 41, 42]. Biochemically, ED cases have been found to have higher serum amyloid A and fibrinogen concentrations compared with healthy horses, co‐grazers, and non‐inflammatory colic cases [43]. Additionally, horses with acute ED have significant increases in plasma taurine concentration, along with variable increases in serum alanine, glutamate, and glycine concentrations and significant decreases in other amino acids, despite expected dehydration‐induced increases [44]. Urinalysis may aid in the diagnosis of ED, with affected horses shown to have significantly higher urine specific gravity, increased protein, creatinine, and glucose concentrations, and a significantly lower pH [45]. Recent research also indicates that ED is linked to decreased diversity in fecal microbiota and altered urinary excretion of specific metabolites, both of which could serve as future targets for ante‐mortem diagnosis [46]. Nasogastric intubation, transrectal palpation, esophagoscopy, and diagnostic imaging findings may reflect abnormal esophageal motility and generalized gastrointestinal ileus [47, 48]. Topical application of dilute phenylephrine eye drops may result in temporary reversal of ptosis in affected horses [49, 50] (Figure 3). Ante‐mortem biopsy techniques have shown diagnostic potential, with B‐amyloid precursor protein immunolabeled rectal biopsy samples aiding diagnosis [41] and tongue papillae histology exhibiting high sensitivity (100%) and specificity (98%) in distinguishing ED cases from control horses [42].

Gross pathologic findings generally reflect the extent of involvement of the enteric nervous system, with chronically affected animals typically being emaciated [32, 48]. Bilateral rhinitis sicca is commonly seen in CGS [51]. Cytological examination of neurons of the cranial cervical ganglion can provide a rapid post‐mortem diagnosis of ED [52] with increased lipofuscin accumulations possibly associated with CGS [53]. A definitive diagnosis of ED is confirmed by histopathologic examination of autonomic ganglia and intestinal enteric nervous system plexuses, with typical histologic features including chromatolysis with loss of Nissl substance, eccentricity or pyknosis of the nuclei, neuronal swelling and vacuolation, accumulation of intracytoplasmic eosinophilic spheroids, and axonal dystrophy [10, 54, 55, 56, 57]. Necropsy of long‐term CGS survivors has identified evidence of substantial neuronal loss in the prevertebral and paravertebral ganglia and the enteric plexuses of the small intestine [37, 55, 58, 59, 60], but the interstitial cells of Cajal networks, the pacemaker cells of the intestine, remain intact, which may contribute to the maintenance of intestinal motility, promoting recovery [37].

**FIGURE 4:**
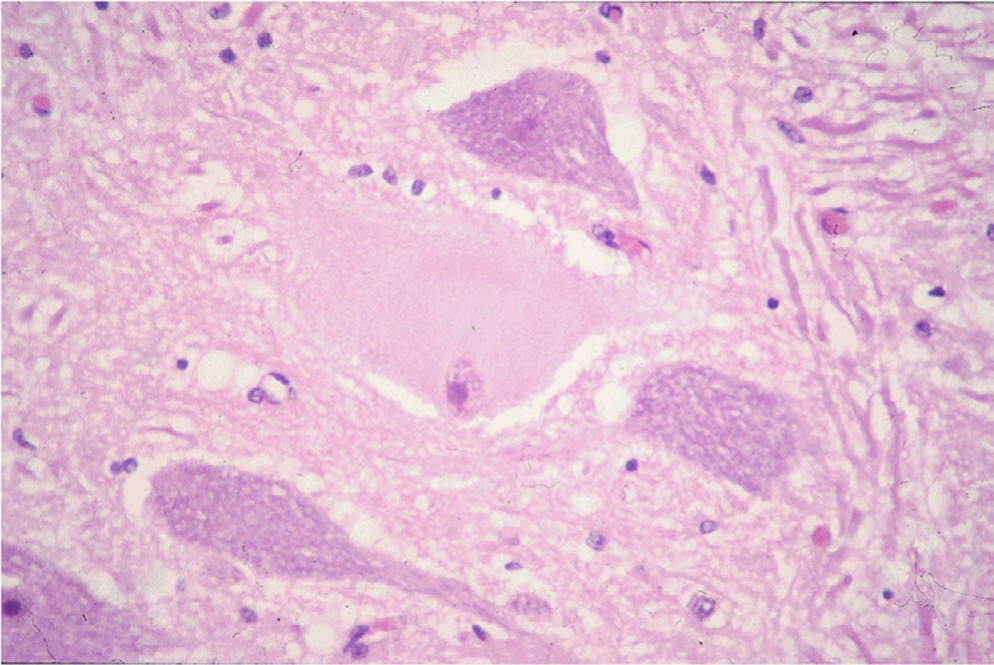
Equine dysautonomia histopathology. Chromatolytic lower motor neuron with characteristic euchromatic nucleus, CN XII, chronic equine grass sickness.

Lesions in the central nervous system consist of neuronal chromatolysis most frequently noted in the autonomic lower motor neurons (LMNs) of the oculomotor nucleus (CN III), parasympathetic nucleus of the vagus nerve (general visceral efferent [GVE] X), and presynaptic LMNs in the intermediolateral horn of the spinal cord (Figure 4). Substantially, the same changes are present in somatic LMN nuclei of CN III, the trigeminal motor nuclei (CN V), facial motor nuclei (CN VII), hypoglossal nuclei (CN XII) and somatic LMNs in the ventral horn of the spinal cord. The prevalence of chromatolysis is found much more often in more chronic cases and in younger horses. The distribution of chromatolytic neurons is unlike that reported in any other disease of horses or humans; however, it appears to be equivalent to that in cats [61], dogs [13], hares [11] and rabbits [62] with primary dysautonomias (Table 1). Unlike the changes seen in the peripheral autonomic neurons, central lesions appear not to be lethal to the individual neuron. Proteomic analysis has identified similarities in neurodegeneration mechanisms between horses with ED and humans, with affected horses showing increased concentrations of proteins typically found in neurodegenerative diseases of humans in their autonomic ganglia [63]. More recently, ED has been linked to structural alterations in skeletal neuromuscular junction ultrastructure characterized by evidence of accelerated synaptic vesicle exocytosis, depletion, and accumulation of neurofilament‐like material, as well as bouton degeneration [64].

**TABLE 1:** Histopathological central nervous system lesions reported in dysautonomias across veterinary species (CN: cranial nerve).

CNS lesion	Equine^1^	Feline^2^	Canine^3^	Leporine^4^	Camelid^5^	Ovine^6^
CN III nuclei	✔	✔	✔	✔		
CN V nuclei	✔	✔		✔		
CN VI nuclei	✔					
CN VII nuclei	✔	✔	✔	✔		
CN VIII nuclei	✔					
Reticular formation	✔					
CN X parasympathetic nucleus	✔	✔	✔	✔		
CN XII nuclei	✔	✔	✔	✔		
Accessory cuneate nucleus					✔	
Sympathetic ganglia		✔			✔	✔
Parasympathetic ganglia		✔				
Somatic peripheral nerves		✔				
Autonomic nerves	✔	✔	✔			
Dorsal root ganglia	✔	✔	✔			
Spinal cord (ventral horn/intermediolateral nucleus)	✔	✔	✔	✔		

*Note:* 1. Hahn, CN, Mayhew IG, de Lahunta A. Central neuropathology of equine grass sickness. *Acta Neuropathologica* 2001;102:153–159. 2. Sharp NJH, Nash AS, Griffiths IR. Feline dysautonomia (the Key‐Gaskell syndrome): a clinical and pathological study of forty cases. *Journal of Small Animal Practice* 1984;25:599–615. 3. Schultze C, Schanen J, Pohlenz J. Canine dysautonomia resembling Key‐Gaskell syndrome in Germany. *Veterinary Record* 1997;141:496–497. 4. Hahn CN, Whitwell KE, Mayhew IG. Central nervous system pathology in cases of leporine dysautonomia. *Veterinary Record* 2001;149:745–746. 5. Kik MJL, van der Hage MH. Cecal impaction due to dysautonomia in a llama ( *Lama glama* ). *Journal of Zoo and Wildlife Medicine* 1999;30:435–438. 6. Pruden SJ, McAllister MM, Schultheiss PC. Abomasal emptying defect of sheep may be an acquired form of dysautonomia. *Veterinary Pathology* 2004;41:164–169.

Despite extensive research efforts and the large number of reported cases, the cause of ED remains elusive, and it appears to be a complex multifactorial condition reliant on ideal conditions. Evidence suggests that the disease is caused by the absorption of a potential neurotoxin from a resilient, non‐infectious organism found in pastures, occurring seasonally. This toxin then spreads throughout the body to multiple neuroanatomical sites, leading to neuronal degeneration characterized by disruptions in cytoplasmic and cytoskeletal proteins, potentially triggering an immunological response [65]. *Clostridium botulinum* has been implicated as a potential causative agent; however, although a clear association exists [66, 67, 68], definitive evidence of a causal association is lacking. Low *C. botulinum* type C antibody concentrations have been significantly associated with increased risk of EGS [68] but, despite corroborating this association, a nationwide field trial in the United Kingdom (UK) failed to identify a significant protective effect of the *C. botulinum* type C vaccine against ED [69]. Moreover, the observation of neuronal degeneration and increased expression of soluble N‐ethylmaleimide‐sensitive factor attachment receptor (SNARE) proteins in the neuronal cell bodies of ED horses, but not in those with botulism, suggests that ED may not be caused by botulinum neurotoxins [70]. Recently, a study identified structural abnormalities in neuromuscular junctions of EGS‐affected horses, including synaptic vesicle depletion, bouton degeneration, and neurofilament accumulation [71]. These findings point more toward an excitatory presynaptic toxin rather than botulinum neurotoxin, which primarily inhibits vesicle release without causing extensive neuromuscular degeneration and supports the hypothesis proposed previously that a soil‐derived phospholipase A2 (PLA2) neurotoxin is a potential cause [72]. Similar to PLA2 toxins in snake venoms, this neurotoxin may damage neuromuscular junctions by degrading nerve terminals and inducing excessive synaptic vesicle release, consistent with other findings [73]. The potential role of grass‐related metabolites such as cyanogens [74] and pasture‐derived mycotoxins also has been proposed [32, 65, 75] and work in Hungary indicates a possible heritable background to the susceptibility to ED [76].

## 5 Feline Dysautonomia

Feline dysautonomia (FD), also known as Key‐Gaskell syndrome, was first described in cats in Scotland in 1982 [2] with many suspected and histologically confirmed cases subsequently identified throughout Europe [12, 77, 78, 79, 80, 81]. Additional cases have been reported in North America, in both native [82, 83, 84, 85] and imported cats known to have traveled to endemic areas [86, 87], and in New Zealand [77, 88], the United Arab Emirates [89] and Brazil [90, 91].

Feline dysautonomia predominantly affects young cats (median age: 3.9 years) [92, 93]. Cases have been reported in indoor‐only cats [84, 90], free‐roaming cats [77, 91], closed cat colonies [78, 81, 94] and littermates [12, 79, 85, 95, 96, 97]. Usually, only one cat is affected in multi‐cat households [84, 93] although multiple in‐contact cases from the same household have been reported [12, 78, 81, 85, 93].

**FIGURE 5:**
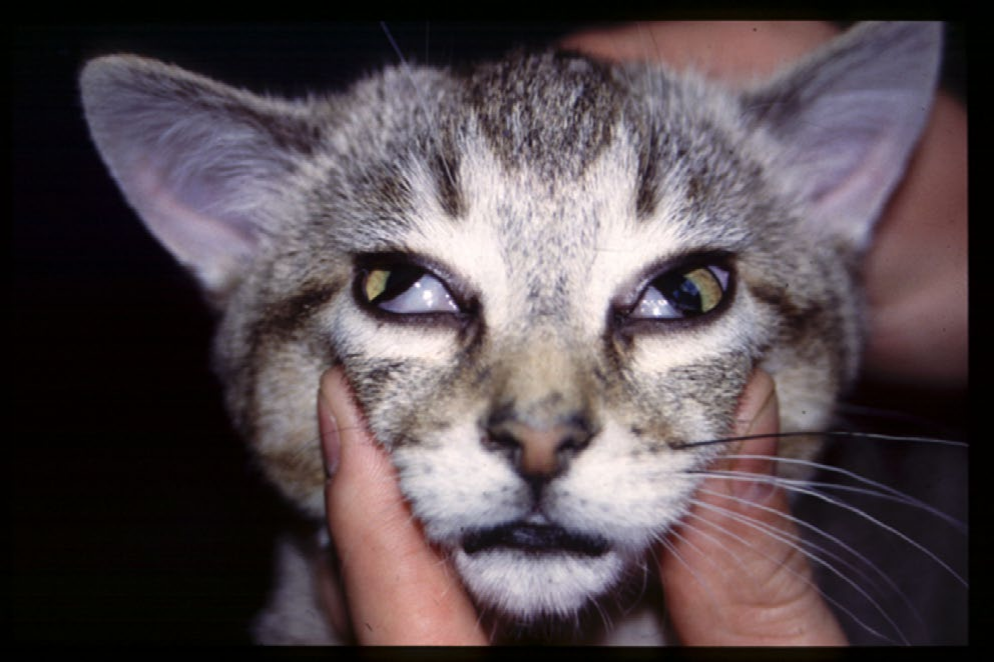
Feline dysautonomia. Cat with feline dysautonomia showing clinical signs of mydriasis and protruding third eyelids.

The most frequent presenting complaints are lethargy, hyporexia or anorexia, and vomiting or regurgitation with constipation, sneezing, dysphagia, stranguria, weight loss, dyspnea, diarrhea, with cervical ventroflexion, and gastro‐esophageal intussusception being less commonly reported [2, 12, 81, 84, 85, 93, 97]. Ocular abnormalities including decreased lacrimation, absent or diminished pupillary light reflex (PLR), bilateral mydriasis, and third eyelid protrusion (Figure 5) are present in over 77% of cases [93, 97] and may be accompanied by abnormalities such as dry mucous membranes, nasal discharge and crusting, and bradycardia [84, 93]. Dysuria, urinary bladder distention, decreased anal tone, and fecal incontinence are less commonly identified [84, 97].

Presumptive diagnosis of FD is based upon clinical signs, physical examination findings, and pharmacologic tests confirming autonomic dysfunction. A positive pilocarpine test response, an absent wheal and flare response to histamine, a poor atropine response test, and imaging findings of megaesophagus, esophageal dysfunction, upper gastrointestinal distension, intestinal ileus, aspiration pneumonia, and megacolon may be suggestive of FD [12, 82, 93, 97, 98, 99].

Feline dysautonomia carries a poor prognosis with survival rates of 10%–50% reported [12, 93, 97]. Cats with milder clinical signs tend to have a better prognosis with long‐term resolution of clinical signs reported [12, 85, 93, 97]. The reason why some cats are more affected than others is unknown. Treatment is symptomatic [93, 100], and definitive diagnosis is made post‐mortem. Gross necropsy examination findings can include megaesophagus and fecal impaction [12]. The histopathological changes and their distribution in cats are strikingly similar to the disease in horses and include chromatolysis, degeneration, and loss of enteric neurons as well as peripheral and central autonomic neurons and, particularly in cases of short duration, neurons in the nuclei of CNs III, V, VII, X, and XII, and spinal cord somatic LMNs with minimal or no inflammatory response [12, 101, 102] (Table 1).

The etiology of the disease is unknown, with infectious, toxic, and metabolic causes proposed. An association with *C. botulinum* neurotoxin has been described [103] and the familial relationships of some affected cats may be suggestive of genetic susceptibility [81]. Some cats diagnosed with FD have decreases in specific detoxifying amino acids, leading to the hypothesis that FD could be the result of ingestion of an unidentified dietary neurotoxic mycotoxin or xenobiotic [104].

## 6 Canine Dysautonomia

Canine dysautonomia (CD) was first reported in England in 1983 [3]. Since then, sporadic individual cases and small case series have been reported across Europe in Scotland [105, 106], Norway [107], Belgium [108, 109], France [110], Germany [13] and England [93, 111, 112, 113]. Interestingly, the majority of confirmed cases are found in the United States (US), a country that does not report the disease in horses, specifically in the Midwest [100, 114, 115, 116, 117, 118]. The first suspected and histologically confirmed cases were reported in Wyoming in 1991 [119] and Missouri in 1996, respectively [114]. Suspected and confirmed cases also have been reported elsewhere in the US and Canada [116, 118, 120, 121].

Canine dysautonomia predominantly affects individual young dogs (median age: 18 months) [114, 116, 117] but there are rare published and unpublished reports of multiple littermates and multi‐dog households affected by the disease [117, 120, 121]. In the US, affected dogs tend to live in rural areas, spending the majority of their time outdoors in close proximity to pasture land, farm ponds, cattle, and wildlife, with most cases identified in the winter and early spring [116, 117].

Clinical signs reflect the severity of neurodegeneration, with the urinary, alimentary, and ocular systems often affected [113, 114, 117]. The most frequent presenting complaints are vomiting, diarrhea, and anorexia [117], and common clinical examination findings are decreased anal tone, absent or diminished PLR, and third eyelid protrusion [113, 117]. Additional findings include weight loss, regurgitation, retching, constipation, dysuria, urinary bladder atony, mydriasis, decreased tear production, dry mucous membranes, and nasal congestion [107, 113, 114, 117, 118]. Other clinical signs suggestive of more extensive nervous system destruction also have been reported [12, 117], including a case report of a Border Collie diagnosed with CD and concurrent autoimmune myasthenia gravis [112]. Paraparesis, loss of anal tone, and fecal incontinence are non‐autonomic findings suggesting somatic motor involvement [100] and may relate to ventral horn cell and dorsal root ganglia lesions [12].

No definitive ante‐mortem test is available and a presumptive diagnosis of CD usually is based upon characteristic multisystem clinical signs and pharmacological tests confirming loss of autonomic function [100, 114]. Decreased lacrimation confirmed with a Schirmer tear test, rapid pilocarpine‐induced miosis [113, 114, 117, 118], a diminished flare response to cutaneous histamine administration, a lack of response of heart rate to atropine administration [118], demonstration of orthostatic hypotension [106, 107, 111] and emptying of an atonic bladder after low‐dose bethanechol administration [114] can facilitate a clinical diagnosis of CD. Radiographic or ultrasonographic evidence of megaesophagus and secondary aspiration pneumonia, gastrointestinal or urinary dysmotility or both, and decreased cardiac contractility also are considered supportive [100, 106, 114, 122, 123]. Ante‐mortem full‐thickness intestinal biopsy demonstrating neuronal degeneration can sometimes confirm the diagnosis of CD [93].

Canine dysautonomia is usually fatal, with most dogs euthanized or dying after the onset of clinical signs [111, 114, 117, 118, 124] and initially only rare (8%–10%) cases of survival were reported [114, 116]. Since 2000, improved survival to discharge (40%–47.5%) and long‐term survival (32%) in suspected CD cases have been reported [93, 113] which may indicate improvement in clinical recognition and symptomatic treatment over time [93, 100, 113]. Necropsy findings reflect the severity of esophageal and gastrointestinal dysmotility, with megaesophagus and aspiration pneumonia frequently reported [100, 114, 125]. Definitive diagnosis of CD requires histological demonstration of the pathognomonic chromatolytic degeneration of the neurons of autonomic ganglia, the ventral horns of the spinal cord, and brainstem nuclei with minimal inflammation [100, 114, 117] (Table 1).

The etiology of CD remains unknown although the regional and temporal proximity of cases suggests that an infectious pathogen, environmental neurotoxin, or food contaminant may be responsible [93, 102, 114, 116, 117, 118, 125]. An autoimmune pathogenesis also has been proposed [112, 116].

## 7 Leporine Dysautonomia

Leporine dysautonomia was first identified in wild hares in East Anglia, England in 1991 shortly after the diagnosis of EGS in horses grazing the same pasture [5]. Subsequent cases have been reported in both wild hares and domestic hares and rabbits elsewhere in the UK [7, 11, 62, 126] and, more recently, in a pet rabbit in Croatia [127]. To our knowledge, dysautonomia has not been identified in North American wildlife. Clinical signs can include cachexia and dehydration [126] but, because of the debilitating nature of the disease, the diagnosis of leporine dysautonomia is usually made post‐mortem [7, 11, 62, 126, 127]. Gross findings include evidence of intestinal stasis, which may be accompanied by inhalation pneumonia and a distended bladder [11, 62]. Definitive diagnosis is confirmed by detecting neuronal degeneration of the postganglionic sympathetic and parasympathetic neurons and chromatolysis of the central nervous system somatic and autonomic LMNs [11, 62] (Table 1). A toxic or dietary cause has been postulated, and botulinum toxin has been confirmed in the gastrointestinal contents of a single case in a wild rabbit [62].

## 8 Camelid Dysautonomia

A dysautonomia syndrome similar to ED has been described in a llama in the Netherlands in 1999 [6] and in a llama and two alpacas in Missouri in 2006 and 2009 [8, 9]. All three presented with an approximately one week‐long history of obstipation, lethargy, and abdominal discomfort [6, 8, 9], with one llama showing clinical signs of generalized autonomic dysfunction including urinary bladder distention, rectal dilatation, and pupillary dysfunction [8]. This llama shared a pasture with healthy miniature horses and another llama that had died after developing similar clinical signs [8]. It is a fatal disease and, in the cases reported, one animal was dead on arrival [6] whereas the other two were promptly euthanized because of lack of improvement or progression of clinical signs [8, 9]. Gross necropsy examination identified intestinal distention in each camelid, which was associated with cecal impaction in one of the llamas and colonic impaction in the alpaca [6, 8, 9]. Histologic abnormalities were similar in each case with neuronal degeneration identified throughout the enteric autonomic nervous system and chromatolysis of neurons of autonomic ganglia and enteric plexi in all three [6, 8, 9] (Table 1). The only central changes described were chromatolysis of the accessory cuneate nucleus in the llama from the Netherlands [6], but the central nervous system of the three cases was not critically studied, and in these cases the accessory cuneate nuclear changes may have been age‐related spheroids. Concurrent salmonellosis (*Salmonella agona*) was confirmed in the esophagus and respiratory tract of the affected llama in Missouri [8].

## 9 Ovine Dysautonomia

Diseases of the autonomic system are uncommon in ruminants [128]. A suspected acquired form of dysautonomia referred to as abomasal emptying defect (AED) has been reported in sheep [129]. This disease, also known as abomasal impaction, was first reported in 1983 [130]. It primarily affects Suffolk sheep [129, 130, 131] but also has been reported in two Hampshire [132], one Dorset [133] and one Texel sheep [134]. The disease is characterized by anorexia and chronic progressive weight loss over weeks to months [131] and distension and impaction of the abomasum [129]. Histological examination of nine necropsy‐confirmed cases of AED in Suffolk and Suffolk‐mix sheep identified scattered chromatolysis and neuronal necrosis in the celiacomesenteric ganglia of six affected sheep, suggestive of neurotoxicosis [129] (Table 1). The cause of AED is unknown [129] but concurrent scrapie has been confirmed in three individual cases [129, 133]. Importantly, examination of the brainstem failed to identify chromatolysis in somatic or autonomic LMNs, which suggests that this syndrome may not be related to the disease described in other animals described in this review.

## 10 Bovine Dysautonomia

Clinical signs consistent with dysautonomia have been recognized in buffalo calves diagnosed with foot‐and‐mouth disease virus in outbreaks in Egypt [135]. Signs of dysautonomia observed before death included partial or complete intestinal dysfunction, loss of anal sphincter tone, tachypnea, body temperature variations, and cardiac arrhythmias [135]. Post‐mortem histopathology of central nervous system tissues identified various degenerative changes with limited or no inflammatory response, but chromatolysis in somatic or autonomic LMNs was not reported [135] which suggests that this syndrome may not be related to the disease described in other animals. Interestingly, incidental central chromatolysis has been identified in bovine ganglia [136]. Although seemingly more common in younger cattle, no obvious association between this histological finding and autonomic nervous system dysfunction was identified, and the changes were as common in clinically normal animals as in diseased animals [136].

## 11 Dysautonomia in Humans

Alpha‐synucleinopathies are neurodegenerative disorders in humans associated with primary dysautonomia [137]. They are characterized by the abnormal aggregation of the protein α‐synuclein within the central and enteric nervous systems [137, 138]. Diseases such as Parkinson's disease, dementia with Lewy bodies, multiple system atrophy, pure autonomic failure (PAF) and rapid‐eye‐movement (REM) sleep behavior disorder fall under this category and are differentiated by the cellular location and pattern of the misfolded α‐synuclein deposition [137, 138].

Pure autonomic failure is characterized by predominant peripheral α‐synuclein deposition in autonomic ganglia and nerves [138]. It is most similar to the dysautonomia reported in veterinary species, but there is overlap with the other major synucleinopathies of humans, and often PAF will progress to a central synucleinopathy with motor or cognitive involvement [137, 138].

Pure autonomic failure, also known as Bradbury‐Eggleston syndrome, was first reported in 1925 [139]. The incidence is unknown, but it is regarded as a rare, sporadic disorder that affects middle‐aged adults, more commonly men, with no known genetic or environmental cause [138, 140]. Neurogenic orthostatic hypotension, with a tendency for syncope, is the hallmark of PAF [139] and results from inadequate vasomotor sympathetic release of epinephrine because of peripheral vasomotor denervation [137]. Other common clinical features include constipation, bladder dysfunction, sexual impotence, thermoregulatory abnormalities, and anosmia [137, 138, 140, 141]. Dream enactment behavior, indicative of REM sleep behavior disorder (a parasomnia characterized by the loss of normal skeletal muscle atonia during REM sleep [142]) is common and suggests central nervous system involvement in PAF [137, 138, 141].

Patients with PAF often exhibit mild anemia, renal dysfunction with proteinuria, cardiac damage, such as left ventricular hypertrophy, and neuroimaging may show cerebrovascular changes linked to blood pressure variability [137, 138, 141, 143, 144, 145]. Because of the dysfunction of peripheral sympathetic nerves, catecholamine studies may identify low supine norepinephrine concentrations [137, 138, 146]. Neuroendocrine studies confirm normal baroreflex function [137]. Urodynamic studies often show detrusor hyperreflexia [137]. It appears from the literature that PAF‐specific objective gastrointestinal studies have not been reported, but esophageal dysmotility, delayed gastric emptying, and slow colonic transit are documented in patients with synucleinopathies [137, 147, 148]. Cardiac sympathetic neuroimaging studies have demonstrated decreased cardiac sympathetic nerve activity [149] and the ability to predict the transformation of PAF into other neurodegenerative conditions [150]. Alpha‐synuclein deposits may be identified in biopsied dermal nerve fibers [138, 151] whereas cerebrospinal fluid assays for α‐synuclein seeding activity and neurofilament light chain concentrations can help predict the progression of PAF to conditions such as multiple system atrophy or Parkinson's disease or dementia with Lewy bodies [137, 152, 153].

There is currently no cure for PAF [137]. Treatment focuses on managing neurogenic orthostatic hypotension through non‐pharmacological and pharmacological means, along with strategies to alleviate other autonomic dysfunction symptoms [137, 138]. Most patients experience slow progression over many years, although approximately one‐third of patients will progress to a central synucleinopathy within four years [137, 138, 141].

Gross necropsy findings are consistent with end organ pathology correlating with sympathetic and parasympathetic failure [154]. Histopathological studies in most PAF patients have identified α‐synuclein deposits and intraneuronal cytoplasmic misfolded α‐synuclein aggregates (Lewy bodies) in the sympathetic ganglia and parasympathetic ganglia of visceral organs [154, 155, 156]. Lewy bodies also have been found in the substantia nigra, the locus coeruleus, and the thoracolumbar and sacral spinal cord, but without associated neuronal loss [138, 154, 155, 156]. A pathologically and neurochemically distinct, non‐Lewy body form of PAF also has been identified [156]. Both PAF phenotypes have a sympathetic preganglionic lesion, but only Lewy body PAF, Parkinson's disease and dementia with Lewy bodies feature a postganglionic noradrenergic lesion [156]. Lewy body PAF may transform into Parkinson's disease or dementia with Lewy bodies, but not into multiple system atrophy, whereas non‐Lewy body PAF can transform into multiple system atrophy [156].

## 12 Conclusion

The etiology of dysautonomia in each species remains elusive, with variable evidence supporting different potential factors. It appears to be a disease requiring specific animal, seasonal, and geographical conditions, but the multiple other factors likely influencing pathological illness have yet to be identified. *C. botulinum* has been implicated [62, 66, 67, 68, 102], but clear causative links are lacking. Other potential sources include environmental neurotoxins [32, 65, 71, 72, 73, 75, 104] such as phospholipase A2 (PLA2) [72, 73], pasture‐related metabolites [74] and even genetic susceptibility [12, 76, 79, 81, 85, 95, 96, 97, 118], indicating that the disease's etiology may be multifactorial. Despite unclear etiology, the similarity in clinical signs and histopathological findings across species suggests a common underlying mechanism affecting the autonomic nervous system, and it is most likely the same disease process with some species differences.

Recent research efforts have focused on understanding the disease at a molecular level, with studies investigating genomics, proteomics, transcriptomics, and metabolomics to identify potential causes and diagnostic markers. Multidisciplinary approaches eventually may lead to a better understanding of the underlying factors contributing to dysautonomia and offering hope for improved diagnostic tests, treatments, and prevention strategies in the future.

Overall, although much remains to be discovered about primary dysautonomia and its various manifestations across species, ongoing research and technological advances hold promise for uncovering the complex factors driving this rare but impactful condition.

## Disclosure

Authors declare no off‐label use of antimicrobials.

## Ethics Statement

Authors declare no institutional animal care and use committee or other approval was needed. Authors declare human ethics approval was not needed.

## Conflicts of Interest

The authors declare no conflicts of interest.

## References

[1] J. F. Tocher , “Grass Sickness in Horses,” Transactions of the Royal Highland Agricultural Society of Scotland 36 (1924): 65–83.

[2] T. Key and C. J. Gaskell , “Puzzling Syndrome in Cats Associated With Pupillary Dilatation,” Veterinary Record 110 (1982): 160. 10.1136/vr.110.7.160 7064339

[3] I. Rochlitz and A. M. Bennett , “Key‐Gaskell Syndrome in a Bitch,” Veterinary Record 112 (1983): 614–615. 10.1136/vr.112.26.614 6879993

[4] A. D. Wales , A. S. Blunden , and O. M. Hosegood , “Grass Sickness With Atypical Presentation in a Young Zebra,” Veterinary Record 148 (2001): 818–819. 11467613 10.1136/vr.148.26.818

[5] K. E. Whitwell , “Do Hares Suffer From Grass Sickness?,” Veterinary Record 128 (1991): 395–396. 1858259 10.1136/vr.128.17.395

[6] M. J. L. Kik and M. H. van der Hage , “Cecal Impaction Due to Dysautonomia in a Llama ( *Lama glama* ),” Journal of Zoo and Wildlife Medicine 30 (1999): 435–438. 10572871

[7] I. R. Griffiths and K. E. Whitwell , “Leporine Dysautonomia: Further Evidence That Hares Suffer From Grass Sickness,” Veterinary Record 132 (1993): 376–377. 8488648 10.1136/vr.132.15.376

[8] J. R. Middleton , G. C. Johnson , I. Pardo , M. Chigerwe , and D. P. O'Brien , “Dysautonomia and Salmonellosis in an 11‐Year‐Old Female Llama ( *Lama glama* ),” Journal of Veterinary Internal Medicine 20 (2006): 213–216. 16496946 10.1892/0891-6640(2006)20[213:dasiay]2.0.co;2

[9] C. A. Lewis , C. C. Bozynski , G. C. Johnson , C. M. Harral , F. Williams , and J. W. Tyler , “Colonic Impaction Due to Dysautonomia in an Alpaca,” Journal of Veterinary Internal Medicine 23 (2009): 1117–1122. 19627474 10.1111/j.1939-1676.2009.0351.x

[10] C. N. Hahn , I. G. Mayhew , and A. de Lahunta , “Central Neuropathology of Equine Grass Sickness,” Acta Neuropathologica 102 (2001): 153–159. 11563630 10.1007/s004010000289

[11] C. N. Hahn , K. E. Whitwell , and I. G. Mayhew , “Central Nervous System Pathology in Cases of Leporine Dysautonomia,” Veterinary Record 149 (2001): 745–746. 11808657

[12] N. J. H. Sharp , A. S. Nash , and I. R. Griffiths , “Feline Dysautonomia (The Key‐Gaskell Syndrome): A Clinical and Pathological Study of Forty Cases,” Journal of Small Animal Practice 25 (1984): 599–615.

[13] C. Schultze , J. Schanen , and J. Pohlenz , “Canine Dysautonomia Resembling Key‐Gaskell Syndrome in Germany,” Veterinary Record 141 (1997): 496–497. 9402721 10.1136/vr.141.19.496

[14] D. F. Cottrell , B. C. McGorum , and G. T. Pearson , “The Neurology and Enterology of Equine Grass Sickness: A Review of Basic Mechanisms,” Neurogastroenterology and Motility 11, no. 2 (1999): 79–92, 10.1046/j.1365-2982.1999.00140.x. 10320588

[15] H. E. McCarthy , C. J. Proudman , and N. P. French , “Epidemiology of Equine Grass Sickness: A Literature Review (1909‐1999),” Veterinary Record 149 (2001): 293–300. 11570789 10.1136/vr.149.10.293

[16] B. Schwarz , R. Brunthaler , C. Hahn , and R. van den Hoven , “Outbreaks of Equine Grass Sickness in Hungary,” Veterinary Record 170 (2012): 75. 22124026 10.1136/vr.100141

[17] B. C. McGorum and R. S. Pirie , “Equine Dysautonomia,” Veterinary Clinics of North America, Equine Practice 34, no. 1 (2018): 113–125, 10.1016/j.cveq.2017.11.010. 29398183

[18] F. Laus , J. Corsalini , and M. T. Mandara , “Equine Grass Sickness in Italy: A Case Series Study,” BMC Veterinary Research 17 (2021): 264. 34362361 10.1186/s12917-021-02966-y PMC8343987

[19] J. A. Woods and J. S. Gilmour , “A Suspected Case of Grass Sickness in The Falkland Islands,” Veterinary Record 128 (1991): 359–360. 2063540 10.1136/vr.128.15.359

[20] W. J. Stewart , “A Case of Suspected Acute Grass Sickness in a Thoroughbred Mare,” Australian Veterinary Journal 53 (1977): 196. 10.1111/j.1751-0813.1977.tb00179.x 869818

[21] F. A. Uzal , C. A. Robles , and F. V. Olaechea , “Histopathological Changes in the Coeliaco‐Mesenteric Ganglia of Horses With ‘Mal Seco’, a Grass Sickness‐Like Syndrome, in Argentina,” Veterinary Record 130 (1992): 244–246. 1285752 10.1136/vr.130.12.244

[22] F. A. Uzal and C. A. Robles , “Mal Seco, A Grass Sickness‐Like Syndrome of Horses in Argentina,” Veterinary Research Communications 17 (1993): 449–457. 8030198 10.1007/BF01839212

[23] F. A. Uzal , D. L. Doxey , C. A. Robles , M. P. Woodman , and E. M. Milne , “Histopathology of the Brain‐Stem Nuclei of Horses With “Mal Seco”, An Equine Dysautonomia,” Journal of Comparative Pathology 111 (1994): 297–301. 7836571 10.1016/s0021-9975(05)80008-8

[24] O. Araya , L. Vits , E. Paredes , and R. Ildefonso , “Grass Sickness in Horses in Southern Chile,” Veterinary Record 150 (2002): 695–697. 12074241 10.1136/vr.150.22.695

[25] A. Wright , L. Beard , B. Bawa , et al., “Dysautonomia in a Six‐Year‐Old Mule in the United States,” Equine Veterinary Journal 42 (2010): 170–173. 20156255 10.2746/042516409X479595

[26] S. J. Girling , M. A. Fraser , D. Richardson , et al., “An Acute Outbreak of Equine Dysautonomia (Equine Grass Sickness) in a Group of Eight Przewalski's Horses ( *Equus ferus* [Caballus] Przewalskii),” Equine Veterinary Journal 29 (2017): 358–361.

[27] N. P. French , H. E. McCarthy , P. J. Diggle , et al., “Clustering of Equine Grass Sickness Cases in the United Kingdom: A Study Considering the Effect of Position Dependent Reporting on the Space‐Time‐K‐Function,” Epidemiology and Infection 133 (2005): 343–348. 15816161 10.1017/s0950268804003322 PMC2870255

[28] J. L. N. Wood , E. M. Milne , and D. L. Doxey , “A Case‐Control Study of Grass Sickness (Equine Dysautonomia) in the United Kingdom,” Veterinary Journal 156 (1998): 7–14. 9691846 10.1016/s1090-0233(98)80055-5

[29] J. S. Gilmour and D. L. Mould , “Experimental Studies of Neurotoxic Activity in Blood Fractions From Acute Cases of Grass Sickness,” Research in Veterinary Science 22 (1977): 1–4. 841191

[30] G. B. Edwards , “Equine Dysautonomia. Grass Sickness of Horses: Clinical Picture and Management,” Journal of Small Animal Practice 28 (1987): 364–368.

[31] D. L. Doxey , J. S. Gilmour , and E. M. Milne , “The Relationship Between Meteorological Features and Equine Grass Sickness (Dysautonomia),” Equine Veterinary Journal 23 (1991): 370–373. 1959529 10.1111/j.2042-3306.1991.tb03740.x

[32] R. S. Pirie , R. C. Jago , and N. P. H. Hudson , “Equine Grass Sickness,” Equine Veterinary Journal 46 (2014): 545–553. 24580639 10.1111/evj.12254

[33] D. L. Doxey , E. M. Milne , and A. Harter , “Recovery of Horses From Dysautonomia (Grass Sickness),” Veterinary Record 137 (1995): 585–588. 8748171

[34] R. C. Jago , I. Handel , C. N. Hahn , et al., “Bodyweight Change Aids Prediction of Survival in Chronic Equine Grass Sickness,” Equine Veterinary Journal 48 (2016): 792–797. 26701780 10.1111/evj.12551

[35] D. L. Doxey , S. Tothill , E. M. Milne , et al., “Patterns of Feeding Behaviour in Horses Recovering From Dysautonomia (Grass Sickness),” Veterinary Record 137 (1995): 181–183. 8560722 10.1136/vr.137.8.181

[36] D. L. Doxey , E. M. Milne , J. Ellison , and P. J. S. Curry , “Long‐Term Prospects for Horses With Grass Sickness (Dysautonomia),” Veterinary Record 142 (1998): 207–209. 9533290 10.1136/vr.142.9.207

[37] E. M. Milne , R. S. Pirie , and C. N. Hahn , “A Study of Residual Lesions in Horses That Recovered From Clinical Signs of Chronic Equine Dysautonomia,” Journal of Veterinary Internal Medicine 33, no. 5 (2019): 2302–2311, 10.1111/jvim.15567. 31332854 PMC6766533

[38] S. F. C. Scholes , C. Vaillant , P. Peacock , G. Edwards , and D. Kelly , “Diagnosis of Grass Sickness by Ileal Biopsy,” Veterinary Record 133 (1993): 7–10. 8362491 10.1136/vr.133.1.7

[39] B. E. Waggett , B. C. McGorum , D. J. Shaw , et al., “Evaluation of Synaptophysin as an Immunohistochemical Marker for Equine Grass Sickness,” Journal of Comparative Pathology 142 (2009): 284–290. 20045117 10.1016/j.jcpa.2009.11.004

[40] A. D. Wales and K. E. Whitwell , “Potential Role of Multiple Rectal Biopsies in the Diagnosis of Equine Grass Sickness,” Veterinary Record 158 (2006): 372–777. 16547184 10.1136/vr.158.11.372

[41] R. C. Jago , S. Scholes , T. S. Mair , et al., “Histological Assessment of b‐Amyloid Precursor Protein Immunolabelled Rectal Biopsies Aids Diagnosis of Equine Grass Sickness,” Equine Veterinary Journal 50 (2018): 22–28. 28621903 10.1111/evj.12710

[42] B. C. McGorum , R. S. Pirie , D. Shaw , N. Macintyre , and A. Cox , “Neuronal Chromatolysis in the Subgemmal Plexus of Gustatory Papillae in Horses With Grass Sickness,” Equine Veterinary Journal 48, no. 6 (2016): 773–778, 10.1111/evj.12530. 26518231

[43] V. E. N. Copas , A. E. Durham , C. H. Stratford , et al., “In Equine Grass Sickness, Serum Amyloid A and Fibrinogen Are Elevated, and Can Aid Differential Diagnosis From Non‐Inflammatory Causes of Colic,” Veterinary Record 172 (2013): 15. 23428423 10.1136/vr.101224

[44] B. C. McGorum and J. C. Kirk , “Equine Dysautonomia (Grass Sickness) is Associated With Altered Plasma Amino Acid Levels and Depletion of Plasma Sulphur Amino Acids,” Equine Veterinary Journal 33 (2001): 473–477. 11558742 10.2746/042516401776254763

[45] C. Fintl , E. M. Milne , and B. C. McGorum , “Evaluation of Urinalysis as an Aid in the Diagnosis of Equine Grass Sickness,” Veterinary Record 151 (2002): 721–724. 12509076

[46] J. Leng , C. Proudman , A. Darby , et al., “Exploration of the Fecal Microbiota and Biomarker Discovery in Equine Grass Sickness,” Journal of Proteome Research 17 (2018): 1120–1128. 29364680 10.1021/acs.jproteome.7b00784

[47] T. R. C. Greet and K. E. Whitwell , “Barium Swallow as an Aid to the Diagnosis of Grass Sickness,” Equine Veterinary Journal 18 (1986): 294–297. 3758008 10.1111/j.2042-3306.1986.tb03633.x

[48] C. Lyle and R. S. Pirie , “Equine Grass Sickness,” In Practice 31 (2009): 26–32.

[49] C. N. Hahn and I. G. Mayhew , “Phenylephrine Eye Drops as a Diagnostic Test in Equine Grass Sickness,” Veterinary Record 147 (2000): 603–606. 11110481 10.1136/vr.147.21.603

[50] C. N. Hahn and I. G. Mayhew , “Studies on the Experimental Induction of Ptosis in Horses,” Veterinary Journal 160 (2000): 220–224. 11061958 10.1053/tvjl.2000.0493

[51] E. M. Milne , M. P. Woodman , and D. L. Doxey , “Use of Clinical Measurements to Predict the Outcome in Chronic Cases of Grass Sickness (Equine Dysautonomia),” Veterinary Record 134 (1994): 438–440. 8048214 10.1136/vr.134.17.438

[52] C. Piccinelli , R. Jago , and E. Milne , “Ganglion Cytology: A Novel Rapid Method for the Diagnosis of Equine Dysautonomia,” Veterinary Pathology 56 (2019): 244–247. 30286693 10.1177/0300985818806051

[53] L. Tan Yi Shean , E. M. Milne , D. J. Shaw , et al., “Lipofuscin Accumulates in Ganglionic Neurons in Chronic Equine Dysautonomia,” Journal of Veterinary Diagnostic Investigation 36, no. 6 (2024): 864–869. 39113499 10.1177/10406387241265715 PMC11529066

[54] A. Obel , “Studies on Grass Disease: The Morphological Picture With Specific Reference to the Vegetative Nervous System,” Journal of Comparative Pathology 65 (1955): 334–346. 13263453

[55] S. F. C. Scholes , C. Vaillant , P. Peacock , G. Edwards , and D. Kelly , “Enteric Neuropathy in Horses With Grass Sickness,” Veterinary Record 132 (1993): 647–651. 8362469 10.1136/vr.132.26.647

[56] A. Brownlee , “Changes in the Coeliaco‐Mesenteric Ganglia of Horses Affected With Grass Sickness and of Horses Affected With Some Other Diseases,” Veterinary Record 71 (1959): 668–669. 14329775

[57] J. S. Gilmour , “Chromatolysis and Axonal Dystrophy in the Autonomic Nervous System in Grass Sickness of Equidae,” Neuropathology and Applied Neurobiology 1 (1975): 39–47.

[58] D. L. Doxey , P. Johnston , C. Hahn , et al., “Histology in Recovered Cases of Grass Sickness,” Veterinary Record 146, no. 22 (2000): 645–646, 10.1136/vr.146.22.645. 10872787

[59] D. M. Pogson , D. L. Doxey , J. S. Gilmour , E. M. Milne , and H. K. Chisholm , “Autonomic Neurone Degeneration in Equine Dysautonomia (Grass Sickness),” Journal of Comparative Pathology 107 (1992): 271–283. 1469124 10.1016/0021-9975(92)90003-d

[60] A. Murray , G. T. Pearson , and D. F. Cottrell , “Light Microscopy of the Enteric Nervous System of Horses With or Without Equine Dysautonomia (Grass Sickness): Its Correlation With the Motor Effects of Physostigmine,” Veterinary Research Communications 21 (1997): 507–520. 9345718 10.1023/a:1005998505369

[61] I. R. Griffiths , A. S. Nash , and N. J. H. Sharp , “The Key‐Gaskell Syndrome: The Current Situation,” Veterinary Record 111 (1982): 532–533. 7179708

[62] C. N. Hahn , K. E. Whitwell , and I. G. Mayhew , “Neuropathological Lesions Resembling Equine Grass Sickness in Rabbits,” Veterinary Record 156 (2005): 778–779. 15951502 10.1136/vr.156.24.778

[63] B. C. McGorum , R. S. Pirie , S. L. Eaton , et al., “Proteomic Profiling of Cranial (Superior) Cervical Ganglia Reveals Beta‐Amyloid and Ubiquitin Proteasome System Perturbations in an Equine Multiple System Neuropathy,” Molecular & Cellular Proteomics 14, no. 11 (2015): 3072–3086, 10.1074/mcp.M115.054635. 26364976 PMC4638047

[64] B. C. McGorum , T. Davey , M. C. M. Dosi , et al., “Equine Grass Sickness Is Associated With Major Abnormalities in the Ultrastructure of Skeletal Neuromuscular Junctions,” Equine Veterinary Journal 57 (2025): 193–202. 38301732 10.1111/evj.14063 PMC11616959

[65] R. S. Pirie and B. C. McGorum , “Equine Grass Sickness: An Update,” UK‐Vet Equine 2 (2018): 6–10.

[66] L. C. Hunter , J. K. Miller , and I. R. Poxton , “The Association of *Clostridium botulinum* Type C With Equine Grass Sickness: A Toxicoinfection,” Equine Veterinary Journal 31 (1999): 492–499. 10596931 10.1111/j.2042-3306.1999.tb03857.x

[67] L. C. Hunter and I. R. Poxton , “Systemic Antibodies to *Clostridium botulinum* Type C: Do They Protect Horses From Grass Sickness (Dysautonomia),” Equine Veterinary Journal 33 (2001): 547–553. 11720025 10.2746/042516401776563418

[68] H. E. McCarthy , N. P. French , G. B. Edwards , et al., “Equine Grass Sickness Is Associated With Low Antibody Levels to *Clostridium botulinum*: A Matched Case‐Control Study,” Equine Veterinary Journal 36, no. 2 (2004): 123–129, 10.2746/0425164044868611. 15038434

[69] J. Ireland , “Nationwide Field Trial of a Candidate Vaccine for the Prevention of Equine Grass Sickness,” Equine Grass Sickness Fund [Internet]. 2019 November, https://www.grasssickness.org.uk/research/results‐of‐a‐nationwide‐field‐trial‐for‐a‐vaccine‐for‐the‐prevention‐of‐equine‐grass‐sickness/.

[70] B. C. McGorum , S. Scholes , E. M. Milne , et al., “Equine Grass Sickness, but Not Botulism, Causes Autonomic and Enteric Neurodegeneration and Increases Soluble N‐Ethylmaleimide‐Sensitive Factor Attachment Receptor Protein Expression Within Neuronal Perikaryal,” Equine Veterinary Journal 48 (2015): 786–791. 10.1111/evj.12543 26640078

[71] B. C. McGorum , T. Davey , M. C. M. Dosi , et al., “Equine Grass Sickness is Associated With Major Abnormalities in the Ultrastructure of Skeletal Neuromuscular Junctions,” Equine Veterinary Journal 57, no. 1 (2025): 193–202, 10.1111/evj.14063. 38301732 PMC11616959

[72] C. Montecucco and L. Bano , “A Possible Cause and Therapy for Equine Grass Sickness,” Toxicon 245 (2024): 107790. 38821320 10.1016/j.toxicon.2024.107790

[73] B. C. McGorum , R. S. Pirie , L. Bano , et al., “Neurotoxic Phospholipase A2: A Proposed Cause of Equine Grass Sickness and Other Animal Dysautonomias (Multiple System Neuropathies),” Equine Veterinary Journal 57 (2025): 11–18. 39630613 10.1111/evj.14442

[74] B. C. McGorum and R. A. Anderson , “Biomarkers of Exposure to Cyanogens in Horses With Grass Sickness,” Veterinary Record 151 (2002): 442–445. 12408327 10.1136/vr.151.15.442

[75] B. C. McGorum , Z. Chen , L. Glendinning , et al., “Equine Grass Sickness (A Multiple Systems Neuropathy) is Associated With Alterations in the Gastrointestinal Mycobiome,” Animal Microbiome 3, no. 1 (2021): 70, 10.1186/s42523-021-00131-2. 34627407 PMC8501654

[76] B. Vincze , M. Varga , O. Kutasi , et al., “Family Aggregation Analysis Shows a Possible Heritable Background of Equine Grass Sickness (Dysautonomia) in a Hungarian Stud Population,” Acta Veterinaria Hungarica 68 (2020): 263–268. 33128520 10.1556/004.2020.00038

[77] A. T. B. Edney and C. J. Gaskell , “Feline Dysautonomia Around the World,” Veterinary Record 123 (1988): 451–452. 10.1136/vr.123.17.451 3201691

[78] H. W. Symonds , P. McWilliams , H. Thompson , et al., “A Cluster of Cases of Feline Dysautonomia (Key‐Gaskell Syndrome) in a Close Colony of Cats,” Veterinary Record 136 (1995): 353–355. 7610539 10.1136/vr.136.14.353

[79] M. M. Goodman , M. Clark , and K. Warner , “Key‐Gaskell Syndrome in Three Cats,” Veterinary Record 143 (1998): 428. 9807798

[80] W. R. Lyons , “Key‐Gaskell Syndrome in Cats,” Veterinary Record 143 (1998): 568. 9854326

[81] T. A. Cave , C. Knottenbelt , D. J. Mellor , F. Nunn , P. Nart , and S. W. J. Reid , “Outbreak of Dysautonomia (Key‐Gaskell Syndrome) in a Closed Colony of Pet Cats,” Veterinary Record 153 (2003): 387–392. 14567662

[82] W. G. Guilford , D. P. O'Brien , A. Allert , and H. M. Ermeling , “Diagnosis of Dysautonomia in a Cat by Autonomic Nervous System Function Testing,” Journal of the American Veterinary Medical Association 193 (1988): 823–828. 3192461

[83] J. K. Levy , K. M. James , L. D. Cowgill , W. G. Guilford , and A. P. Davidson , “Decreased Urinary Catecholamines in a Cat With Dysautonomia,” Journal of the American Veterinary Medical Association 205 (1994): 842–844. 7829377

[84] A. C. Kidder , C. Johannes , D. P. O'Brien , K. R. Harkin , and T. Schermerhorn , “Feline Dysautonomia in the Midwestern United States: A Retrospective Study of Nine Cases,” Journal of Feline Medicine and Surgery 10 (2008): 130–136. 17950646 10.1016/j.jfms.2007.08.005 PMC10911208

[85] P. Černá , M. M. Botts , A. Watson , et al., “Dysautonomia in Two Littermate Kittens,” Journal of Feline Medicine and Surgery Open Reports 9 (2023), 10.1177/20551169231164. PMC10155007 37151741

[86] N. M. Bromberg and L. D. Cabaniss , “Feline Dysautonomia: A Case Report,” Journal of the American Animal Hospital Association 24 (1988): 106–109.

[87] D. D. Canton , N. J. H. Sharp , and G. D. Aguirre , “Dysautonomia in a Cat,” Journal of the American Veterinary Medical Association 192 (1988): 1293–1296. 3391854

[88] H. J. Beban , R. L. Beban , R. G. Lindsay , and H. P. Bentall , “A Suspected Case of Feline Dysautonomia,” New Zealand Veterinary Journal 35, no. 4 (1987): 58, 10.1080/00480169.1987.35381. 16031375

[89] C. J. Gaskell and A. T. B. Edney , “Feline Dysautonomia Distribution,” Veterinary Record 117 (1985): 395. 10.1136/vr.117.15.395-b 4060553

[90] B. B. J. Torres , G. C. Martins , P. E. Ferian , B. C. Martins , M. A. Rachid , and E. G. Melo , “Key‐Gaskell Syndrome in Brazil: First Case Report,” Brazilian Journal of Veterinary and Animal Sciences 66 (2014): 1046–1050.

[91] G. Schaefer , D. G. Gerardi , N. B. Castro , et al., “Megaesophagus Secondary to Feline Dysautonomia (Key‐Gaskell Syndrome) in a Cat,” Acta Scientiae Veterinariae 44 (2016): 173.

[92] A. T. B. Edney , C. J. Gaskell , and N. J. H. Sharp , “Waltham Symposium No. 6 Feline Dysautonomia ‐ An Emerging Disease,” Journal of Small Animal Practice 28 (1987): 333–416.

[93] K. E. Clarke , S. Sorrell , C. Breheny , et al., “Dysautonomia in 53 Cats and Dogs: Retrospective Review of Clinical Data and Outcome,” Veterinary Record 187 (2020): e118. 32253356 10.1136/vr.105258

[94] A. S. Nash , H. Thompson , N. Rosengurt , H. Symonds , C. Gaskell , and K. Hughes , “Feline Dysautonomia in Group‐Housed Cats,” Veterinary Record 134 (1994): 175–176. 10.1136/vr.134.7.175 8160340

[95] J. C. Power and J. D. Temple , “Key‐Gaskell Syndrome,” Veterinary Record 111 (1982): 540. 7179709

[96] K. C. Barnett , “Observations on the Feline Dilated Pupil Syndrome,” Veterinary Record 114 (1984): 351. 6719792 10.1136/vr.114.14.351

[97] I. Rochlitz , “Feline Dysautonomia (The Key‐Gaskell or Dilated Pupil Syndrome): A Preliminary Review,” Journal of Small Animal Practice 25 (1984): 587–598.

[98] N. J. Sharp , “Feline dysautonomia,” Seminars in Veterinary Medicine and Surgery (Small Animal) 5 (1990): 67–71. 2191396

[99] R. Novellas , K. E. Simpson , D. A. Gunn‐Moore , and G. J. C. Hammond , “Imaging Findings in 11 Cats With Feline Dysautonomia,” Journal of Feline Medicine and Surgery 12 (2010): 584–591. 20452794 10.1016/j.jfms.2010.01.012 PMC10911480

[100] D. P. O'Brien and G. C. Johnson , “Dysautonomia and Autonomic Neuropathies,” Veterinary Clinics of North America, Small Animal Practice 32 (2002): 251–265. 11785731 10.1016/s0195-5616(03)00087-1

[101] F. Guscetti , A. Pospischil , C. Läuchli , et al., “Pathomorphologie der Felinen Dysautonomie (Key‐Gaskell‐Syndrom). Histologische, Elektronenmikroskopische Und Immunhistologische Befunde Bei 4 Katzen [Pathomorphology of Feline Dysautonomia (Key‐Gaskell Syndrome). Histologic, Electron Microscopic and Immunohistologic Findings in 4 Cats],” Tierärztliche Praxis 19 (1991): 296–301. 1887443

[102] M. M. Pollin and I. R. Griffiths , “A Review of the Primary Dysautonomias of Domestic Animals,” Journal of Comparative Pathology 106 (1992): 99–119. 1597536 10.1016/0021-9975(92)90041-r

[103] F. Nunn , T. A. Cave , C. Knottenbelt , et al., “Association Between Key‐Gaskell Syndrome and Infection by *Clostridium botulinum* Type C/D,” Veterinary Record 155, no. 4 (2004): 111–115, 10.1136/vr.155.4.111. 15328740

[104] B. C. McGorum , H. W. Symonds , C. Knottenbelt , et al., “Alterations in Amino Acid Status in Cats With Feline Dysautonomia,” PLoS One 12, no. 3 (2017): e0174346, 10.1371/journal.pone.0174346. 28333983 PMC5363954

[105] M. Pollin and M. Sullivan , “A Canine Dysautonomia Resembling the Key‐Gaskell Syndrome,” Veterinary Record 118 (1986): 402–403. 3716096 10.1136/vr.118.14.402

[106] P. M. Jamieson , C. L. Scudamore , C. E. Ruppert , et al., “Canine Dysautonomia: Two Clinical Cases,” Journal of Small Animal Practice 42 (2002): 22–26. 10.1111/j.1748-5827.2002.tb00005.x 11833820

[107] J. Presthus and I. Bjerkås , “Canine Dysautonomia in Norway,” Veterinary Record 120 (1987): 463–464. 3603990 10.1136/vr.120.19.463

[108] E. Schrauwen , L. Van Ham , and T. Maenhout , “Canine Dysautonomia: A Case Report,” Veterinary Record 128, no. 5 (1991): 524–552. 1866883 10.1136/vr.128.22.524

[109] E. Schrauwen , “Canine Dysautonomia: Another Case Report,” Veterinary Record 132 (1993): 663–664. 10.1136/vr.132.26.663 8362478

[110] H. Bossy , “Deux cas de dysautonomie canine,” Le Point Vétérinaire 24 (1993): 637–641.

[111] S. J. M. Niessen , J. Eastwood , J. B. A. Smyth , et al., “Five Cases of Canine Dysautonomia in England (2004 to 2006),” Journal of Small Animal Practice 48 (2007): 346–352. 17425697 10.1111/j.1748-5827.2006.00263.x

[112] I. Gajanayake , S. J. M. Niessen , G. B. Cherubini , and G. Diane Shelton , “Autoimmune Myasthenia Gravis and Dysautonomia in a Dog,” Journal of Small Animal Practice 49 (2008): 593–595. 18684149 10.1111/j.1748-5827.2008.00580.x

[113] M. D. Johnson , A. J. Rankin , and J. M. Meekins , “Diminished Pupillary Light Reflexes, Elevated Third Eyelids, and Decreased Tear Production Are Commonly Associated With Canine Dysautonomia,” Journal of the American Veterinary Medical Association 28 (2023): 1–6. 10.2460/javma.23.03.0134 37380162

[114] R. C. Longshore , D. P. O'Brien , G. C. Johnson , A. M. Grooters , and R. A. Kroll , “Dysautonomia in Dogs: A Retrospective Study,” Journal of Veterinary Internal Medicine 10 (1996): 103–109. 8743207 10.1111/j.1939-1676.1996.tb02040.x

[115] D. I. Mawby and K. A. Brenneman , “Dysautonomia in a Mixed Breed Dog,” Veterinary Medicine 92 (1997): 889–894.

[116] R. D. Berghaus , D. P. O'Brien , G. C. Johnson , and J. G. Thorne , “Risk Factors for Development of Dysautonomia in Dogs,” Journal of the American Veterinary Medical Association 218 (2001): 1285–1290. 11330614 10.2460/javma.2001.218.1285

[117] K. R. Harkin , G. A. Andrews , and J. C. Nietfeld , “Dysautonomia in Dogs: 65 Cases (1993‐2000),” Journal of the American Veterinary Medical Association 220 (2002): 633–639. 12418523 10.2460/javma.2002.220.633

[118] N. C. Hull , D. O'Toole , M. M. Miller , et al., “Canine Dysautonomia in a Litter of Havanese Puppies,” Journal of Veterinary Diagnostic Investigation 27, no. 5 (2015): 627–631, 10.1177/1040638715595838. 26179098

[119] L. A. Wise and M. R. Lappin , “A Syndrome Resembling Feline Dysautonomia (Key‐Gaskell Syndrome) in a Dog,” Journal of the American Veterinary Medical Association 198 (1991): 2103–2106. 1679426

[120] D. Caines , L. P. Chantale , S. Kruth , et al., “Autonomic Dysfunction in a Jack Russell Terrier,” Canadian Veterinary Journal 52 (2011): 297–299. PMC3039902 21629424

[121] K. R. Harkin , J. Nietfeld , and J. R. Fischer , “Dysautonomia in a Family of German Shorthaired Pointers,” Journal of the American Animal Hospital Association 38 (2002): 55–59. 11804316 10.5326/0380055

[122] D. A. Detweiler , D. S. Biller , J. J. Hoskinson , and K. R. Harkin , “Radiographic Findings of Canine Dysautonomia in Twenty‐Four Dogs,” Veterinary Radiology & Ultrasound 42 (2001): 108–112. 11327357 10.1111/j.1740-8261.2001.tb00912.x

[123] K. R. Harkin , B. J. Bulmer , and D. S. Biller , “Echocardiographic Evaluation of Dogs With Dysautonomia,” Journal of the American Veterinary Medical Association 235 (2009): 1456–1461. 20001776 10.2460/javma.235.12.1431

[124] R. D. Berghaus , D. P. O'Brien , J. G. Thorne , et al., “Incidence of Canine Dysautonomia in Missouri, USA, Between January 1996 and December 2000,” Preventive Veterinary Medicine 54 (2007): 291–300. 10.1016/s0167-5877(02)00030-2 12163247

[125] L. A. Wise and M. R. Lappin , “Canine Dysautonomia,” Seminars in Veterinary Medicine and Surgery (Small Animal) 5 (1990): 72–74. 2191397

[126] K. E. Whitwell , “More Cases of Leporine Dysautonomia,” Veterinary Record 134 (1994): 223–224. 10.1136/vr.134.9.223 8171817

[127] D. Huber , A. G. Kurilj , and I. C. Šoštarić‐Zuckermann , “A Case of Leporine Dysautonomia From Croatia,” Acta Veterinaria 72 (2022): 118–123.

[128] J. W. Finnie , P. A. Windsor , and A. E. Kessell , “Neurological Diseases of Ruminant Livestock in Australia. I: General Neurological Examination, Necropsy Procedures and Neurological Manifestations of Systemic Disease, Trauma and Neoplasia,” Australian Veterinary Journal 89 (2011): 243–246. 21696371 10.1111/j.1751-0813.2011.00792.x

[129] S. J. Pruden , M. M. McAllister , and P. C. Schultheiss , “Abomasal Emptying Defect of Sheep May Be an Acquired Form of Dysautonomia,” Veterinary Pathology 41 (2004): 164–169. 15017030 10.1354/vp.41-2-164

[130] E. E. Kline , J. R. Meyer , D. R. Nelson , and M. Memon , “Abomasal Impaction in Sheep,” Veterinary Record 113 (1983): 177–179. 6194609 10.1136/vr.113.8.177

[131] P. L. Ruegg , L. W. George , and N. E. East , “Abomasal Dilatation and Emptying Defect in a Flock of Suffolk Ewes,” Journal of the American Veterinary Medical Association 193 (1988): 1534–1536. 3215813

[132] R. M. Smith , W. J. Underwood , G. C. Petersen , and R. Harms , “Abomasal Dilatation and Impaction in Two Hampshire Rams,” Veterinary Record 130 (1992): 468–470. 1626357 10.1136/vr.130.21.468

[133] K. Gabb , J. Lofstedt , and R. Bildfell , “Abomasal Emptying Defect in a Ewe of Predominantly Dorset Breeding,” Veterinary Record 131 (1992): 127–128. 1529516 10.1136/vr.131.6.127

[134] M. W. Sharp and D. F. Collings , “Ovine Abomasal Enlargement and Scrapie,” Veterinary Record 120 (1987): 215. 3576941 10.1136/vr.120.9.215

[135] Y. Bayoumi , N. Sobhy , A. Morsi , W. el‐Neshwey , N. el‐Seddawy , and A. Abdallah , “Clinical and Histopathological Studies on Neurodegeneration and Dysautonomia in Buffalo Calves During Foot‐And‐Mouth Disease Outbreaks in Egypt,” Veterinary World 14 (2021): 1622–1630. 34316212 10.14202/vetworld.2021.1622-1630 PMC8304408

[136] H. Jahns and C. Fast , “A Histopathological Study of Bovine Ganglia,” Journal of Comparative Pathology 150 (2014): 234–244. 24456750 10.1016/j.jcpa.2013.11.207 PMC7094613

[137] G. Lamotte and W. Singer , “Synucleinopathies,” in Handbook of Clinical Neurology, vol. 196 (Elsevier, 2023), 175–202. 37620069 10.1016/B978-0-323-98817-9.00032-6

[138] A. Pavy‐Le Traon , A. Foubert‐Samier , and M. Fabbri , “An Overview on Pure Autonomic Failure,” Revue Neurologique 180 (2024): 94–100. 38129276 10.1016/j.neurol.2023.11.003

[139] E. A. Coon , W. Singer , and P. A. Low , “Pure Autonomic Failure,” Mayo Clinic Proceedings 94 (2019): 2087–2098. 31515103 10.1016/j.mayocp.2019.03.009 PMC6826339

[140] S. Bradbury and C. Eggleston , “Postural Hypotension: A Report of Three Cases,” American Heart Journal 1 (1925): 73–86.

[141] H. Kaufmann , L. Norcliffe‐Kaufmann , J. A. Palma , et al., “Natural History of Pure Autonomic Failure: A United States Prospective Cohort,” Annals of Neurology 81 (2017): 287–297. 28093795 10.1002/ana.24877 PMC5323269

[142] B. F. Boeve , M. H. Silber , C. B. Saper , et al., “Pathophysiology of REM Sleep Behaviour Disorder and Relevance to Neurodegenerative Disease,” Brain 130 (2007): 2770–2788. 17412731 10.1093/brain/awm056

[143] E. M. Garland , A. Gamboa , L. Okamoto , et al., “Renal Impairment of Pure Autonomic Failure,” Hypertension 54 (2009): 1057–1061. 19738158 10.1161/HYPERTENSIONAHA.109.136853 PMC2796115

[144] V. Milazzo , S. Maule , C. Di Stefano , et al., “Cardiac Organ Damage and Arterial Stiffness in Autonomic Failure: Comparison With Essential Hypertension,” Hypertension 66 (2015): 1168–1175. 26459422 10.1161/HYPERTENSIONAHA.115.05913

[145] W. Struhal , H. Lahrmann , and C. J. Mathias , “Incidence of Cerebrovascular Lesions in Pure Autonomic Failure,” Autonomic Neuroscience 179 (2013): 159–162. 23706609 10.1016/j.autneu.2013.04.006

[146] R. J. Polinsky , I. J. Kopin , M. H. Ebert , et al., “Pharmacologic Distinction of Different Orthostatic Hypotension Syndromes,” Neurology 31 (1981): 1. 10.1212/wnl.31.1.1 7192816

[147] I. Claus , J. Suttrup , P. Muhle , et al., “Subtle Esophageal Motility Alterations in Parkinsonian Syndromes: Synucleinopathies vs. Tauopathies,” Movement Disorders Clinical Practice 5, no. 4 (2018): 406–412, 10.1002/mdc3.12616. 30363410 PMC6174416

[148] I. F. Ivan , V. L. Irincu , Ș. Diaconu , et al., “Gastro‐Intestinal Dysfunctions in Parkinson's Disease,” Experimental and Therapeutic Medicine 22 (2021): 1083. 34447476 10.3892/etm.2021.10517 PMC8355716

[149] D. S. Goldstein and Y. Sharabi , “The Heart of PD: Lewy Body Diseases as Neurocardiologic Disorders,” Brain Research 1702 (2019): 74–84. 29030055 10.1016/j.brainres.2017.09.033 PMC10712237

[150] A. Lenka , R. Isonaka , C. Holmes , and D. S. Goldstein , “Cardiac ^18^F‐Dopamine Positron Emission Tomography Predicts the Type of Phenoconversion of Pure Autonomic Failure,” Clinical Autonomic Research 33 (2023): 737–747. 37843677 10.1007/s10286-023-00987-1

[151] V. Donadio , A. Incensi , P. Cortelli , et al., “Skin Sympathetic Fiber α‐Synuclein Deposits: A Potential Biomarker for Pure Autonomic Failure,” Neurology 80 (2013): 725–732. 23390175 10.1212/WNL.0b013e3182825127

[152] M. Rossi , N. Candelise , S. Baiardi , et al., “Ultrasensitive RT‐QuIC Assay With High Sensitivity and Specificity for Lewy Body‐Associated Synucleinopathies,” Acta Neuropathologica 140 (2020): 49–62. 32342188 10.1007/s00401-020-02160-8 PMC7299922

[153] W. Singer , A. M. Schmeichel , M. Shahnawaz , et al., “Alpha‐Synuclein Oligomers and Neurofilament Light Chain Predict Phenoconversion of Pure Autonomic Failure,” Annals of Neurology 89 (2021): 1212–1220. 33881777 10.1002/ana.26089 PMC8168720

[154] K. Hague , P. Lento , S. Morgello , S. Caro , and H. Kaufmann , “The Distribution of Lewy Bodies in Pure Autonomic Failure: Autopsy Findings and Review of the Literature,” Acta Neuropathologica 94 (1997): 192–196. 9255396 10.1007/s004010050693

[155] H. Kaufmann , K. Hague , and D. Perl , “Accumulation of Alpha‐Synuclein in Autonomic Nerves in Pure Autonomic Failure,” Neurology 56 (2001): 980–981. 11294945 10.1212/wnl.56.7.980

[156] D. S. Goldstein , R. Isonaka , G. Lamotte , et al., “Different Phenoconversion Pathways in Pure Autonomic Failure With Versus Without Lewy Bodies,” Clinical Autonomic Research 31 (2021): 677–684. 34669076 10.1007/s10286-021-00829-y PMC10680053

